# Increased Anxiety-Like Behavior and Enhanced Synaptic Efficacy in the Amygdala of GluR5 Knockout Mice

**DOI:** 10.1371/journal.pone.0000167

**Published:** 2007-01-24

**Authors:** Long-Jun Wu, Shanelle W. Ko, Hiroki Toyoda, Ming-Gao Zhao, Hui Xu, Kunjumon I. Vadakkan, Ming Ren, Eva Knifed, Fanny Shum, Jessica Quan, Xue-Han Zhang, Min Zhuo

**Affiliations:** Department of Physiology, Faculty of Medicine, University of Toronto, Toronto, Ontario, Canada; Vrije University Amsterdam, Netherlands

## Abstract

GABAergic transmission in the amygdala modulates the expression of anxiety. Understanding the interplay between GABAergic transmission and excitatory circuits in the amygdala is, therefore, critical for understanding the neurobiological basis of anxiety. Here, we used a multi-disciplinary approach to demonstrate that GluR5-containing kainate receptors regulate local inhibitory circuits, modulate the excitatory transmission from the basolateral amygdala to the central amygdala, and control behavioral anxiety. Genetic deletion of GluR5 or local injection of a GluR5 antagonist into the basolateral amygdala increases anxiety-like behavior. Activation of GluR5 selectively depolarized inhibitory neurons, thereby increasing GABA release and contributing to tonic GABA current in the basolateral amygdala. The enhanced GABAergic transmission leads to reduced excitatory inputs in the central amygdala. Our results suggest that GluR5 is a key regulator of inhibitory circuits in the amygdala and highlight the potential use of GluR5-specific drugs in the treatment of pathological anxiety.

## Introduction

Anxiety is thought to be a way of controlling an animal's response to threatening or potentially threatening stimuli, whereas excessive levels of anxiety, or pathological anxiety, cause distress and suffering [1], [2]. The amygdala is a key circuit for processing neuronal inputs from other parts of the brain, initiating output signals to responding nuclei and generating various physiological responses, including behavioral, autonomic, and hormonal responses related to anxiety [3], [4]. In both humans and animals, activation of the amygdala elicits anxiety whereas lesion of the amygdala impairs the perception of fear [5]. Anatomically, projecting pathways from the thalamus and cerebral cortex terminate at the lateral amygdala and basolateral amygdala (BLA). The pyramidal cells, which are primarily glutamatergic neurons, then relay information to the central amygdala (CeM) [6]. Local interneurons that contain GABA are critical in processing incoming information in the BLA [7]. Efferents from the CeM then go to the periaqueductal gray, brainstem, and hypothalamus and induce anxiety-related behavioral, autonomic and hormonal responses [6], [7].

γ-Aminobutyric acid (GABA) is the primary inhibitory neurotransmitter in the adult mammalian brain, including the amygdala [7]. GABA_A_ receptors, a family of ligand-gated chloride ion channels, mediate the effects of GABA in anxiety-related behaviors [8]–[10]. Deficiencies in GABA_A_ receptors have been implicated in anxiety-like behavior in mice [11]. GABA_A_ receptors are the prime target for benzodiazepines, such as diazepam, that alleviate anxiety in patients [12]. GABA release is important in maintaining inhibitory tone, which also plays a role in anxiety. For example, neuroimaging studies have demonstrated a clear reduction of GABA levels in the occipital cortex of patients with panic disorders [13]. Additionally, glutamate decarboxylase 65 knockout mice displayed increased anxiety-like behavior and reduced GABA levels [14].

Inhibitory influence onto excitatory pyramidal cells, including neurosteroid sensitive tonic inhibition, is involved in anxiety [15], [16]. A balance between excitatory and inhibitory transmission is critical for normal brain function. Hyperexcitation due to enhanced excitatory transmission or reduced inhibitory transmission can promote anxiety-like behavior [7]. In order to maintain such a fine balance, glutamate influences inhibitory transmission through receptors expressed at presynaptic terminals [17] as well as on the postsynaptic membranes of inhibitory synapses [18]. Kainate (KA) receptors, a member of the ionotropic glutamate receptor family, have been intensely investigated in the hippocampus, cortex and spinal cord [19], [20]. The KA receptor family is composed of five different subunits, namely GluR5, GluR6, GluR7, KA1 and KA2, which can form a variety of homomeric and heteromeric receptors [21]. KA receptors are widely distributed in the peripheral and central nervous systems and, in particular, the GluR5 subunit is highly expressed in dorsal root ganglion neurons, piriform and cingulate cortices, hippocampal interneurons, as well as in the BLA [22]–[24]. Recently, it was shown that GluR5 regulates GABAergic transmission in the BLA [25]. Moreover, GluR5 also partly mediates synaptic responses, as well as some forms of synaptic plasticity, in the BLA [22], [26]. What is currently unknown, however, is the functional role of GluR5 in network excitability of the amygdaloid circuitry and behavioral anxiety.

Here we provide the first evidence implicating GluR5 in the expression of anxiety-like behavior. We show that GluR5 knockout (GluR5^−/−^) mice exhibit a significant increase in anxious behavior and that local antagonism of GluR5 in the BLA of wild-type mice could mimic the anxious phenotype of GluR5^−/−^ mice. We further show that GluR5 regulates GABAergic transmission, neuronal excitability and output to the CeM. Our results suggest that the impairment in GABAergic transmission found in GluR5^−/−^ mice affects information integration in the amygdala. This impairment may underlie the increased expression of anxiety in GluR5^−/−^ mice.

## Results

### GluR5^−/−^ Mice Exhibit Increased Anxiety-like Behavior

**Figure 1:**
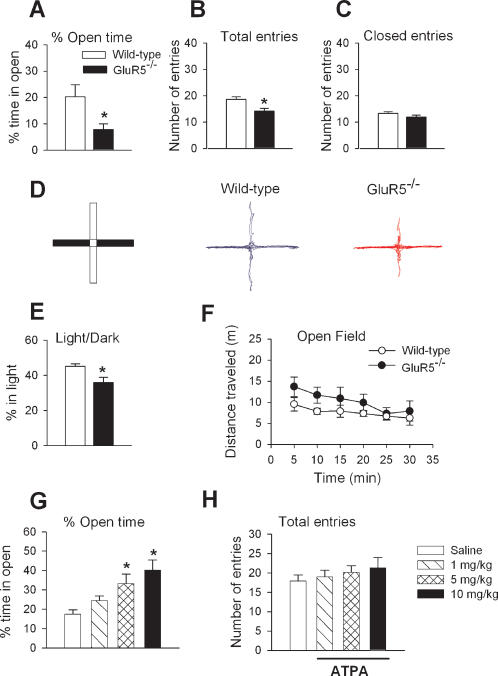
Anxiety-like behavior in GluR5^−/− ^mice. (A) GluR5^−/−^ mice (n = 17) spent significantly less time in the open arms of the EPM compared to wild-type mice (n = 10). (B) The total number of arm entries (open+closed) was decreased in GluR5^−/−^ mice but there was no difference in closed arm entries (C) between genotypes. (D) From left to right: Diagram of the EPM, filled boxes = closed arms, open boxes = open arms; representative traces showing the movement of wild-type and GluR5^−/−^ mice in the EPM for 5 mins. (E) GluR5^−/−^ mice (n = 8) spent significantly less time in the light half of the chamber compared to wild-type mice (n = 6). (F) No significant difference was detected in the distance traveled in the open field. * P<0.05. (G) While open arm exploration was not affected by 1 mg/kg ATPA (n = 5), 5.0 mg/kg (n = 7) and 10.0 mg/kg (n = 10) ATPA significantly increased the time spent in the open arms compared to mice receiving saline (n = 9). (H) There was no difference in the number of total arm entries across treatment groups.

To determine if GluR5 plays a role in the expression of anxiety GluR5^−/− ^(n = 17) and wild-type littermate (n = 10) mice were tested on the elevated plus maze (EPM), a widely used model for anxiety-like behavior. We found that GluR5^−/−^ mice spent significantly less time in the open arms of the EPM, as compared to wild-type animals (P<0.05, Figure 1A). There was also a statistically significant decrease in the number of total arm entries (open + closed) between genotypes (P<0.05, Figure 1B). This decrease was due to a significant reduction in open arm entries (P<0.05) since there was no difference in the total number of closed arm entries (P = 0.24, Figure 1C). The decrease in open arm exploration suggests that GluR5^−/−^ mice have increased anxious behavior in the EPM (Figure 1A and D).

In order to confirm that GluR5^−/−^ mice exhibit enhanced anxiety levels, we conducted a second behavioral test for anxiety, the light/dark exploration test. Our results showed that GluR5^−/−^ mice (n = 8) spent significantly less time exploring the light half of the chamber when compared to wild-type mice (n = 6, P<0.05, Figure 1E). To test for changes in mobility, motor coordination was measured on a RotaRod and locomotor activity was recorded in an open field. We found no significant differences between wild-type and GluR5^−/−^ mice in RotaRod performance (wild-type, n = 6; GluR5^−/−^, n = 10, P = 0.85) or in the total distance traveled within the open field for 30 mins (wild-type, n = 7; GluR5^−/−^, n = 8, P = 0.22) (Figure 1F). Taken together, these results provide strong evidence for an increase in anxiety-like behavior in GluR5^−/−^ mice.

### Activation of GluR5 Receptors Decreases Anxiety in Wild-type Mice

Since the deletion of GluR5 resulted in an increase in anxious behavior, we next wanted to determine if activation of GluR5 would have the opposite effect on anxiety. The selective GluR5 agonist, ATPA, was administered via intraperitoneal injection to wild-type mice 30 minutes before testing in the EPM. There was a significant effect of ATPA on open arm exploration in the EPM (P<0.05, Figure 1G). Animals treated with 5 mg/kg (n = 5, P<0.05) or 10.0 mg/kg (n = 10, P<0.01) ATPA spent significantly more time in the open arms compared to mice receiving saline (n = 9). ATPA did not affect overall exploratory activity since there was no difference in the number of total arm entries across treatment groups (P = 0.68, Figure 1H). Taken together, our results show that the deletion of GluR5 increases anxious behavior in the EPM (Figure 1A) while activation of GluR5 by ATPA decreases anxiety (Figure 1G).

### Increased Anxious Behavior by Local Inhibition of GluR5 in the BLA

**Figure 2:**
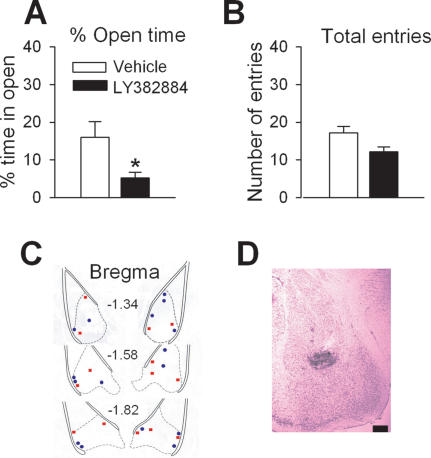
Pharmacological inhibition of GluR5 receptors in the amygdala affected behavioral anxiety. (A) Mice injected with LY382884 (n = 7, 4 µg/µl) spent significantly less time in the open arms of the EPM compared to vehicle injected mice (n = 8). (B) There was no difference in the number of total arm entries between groups. (C) Cannula tip placement in mice injected with Vehicle (blue circles) or LY382884 (red squares) in the BLA. (D) Representative coronal section showing the BLA injection site (scale bar 0.6 mm). * P<0.05.

Since the amygdala is an essential component of the circuitry underlying anxious behavior, we decided to focus on the role of GluR5 receptors in the amygdala, with particular emphasis on the BLA. The selective GluR5 antagonist LY382884 was locally injected into the BLA of wild-type mice and performance on the EPM was evaluated. Mice injected with LY382882 (4 µg/µl, n = 7) spent significantly less time exploring the open arms of the EPM compared to mice receiving the vehicle (n = 8, P<0.05, Figure 2A). There was not a significant difference in total arm entries between treatment groups (P>0.05, Figure 2B) suggesting that intra-amygdalar injection of LY382884 did not affect overall exploratory activity. This result suggests that the anxiety phenotype of GluR5^−/−^ mice can be mimicked by the local antagonism of GluR5 in the BLA.

### Activation of GluR5 Depolarized Interneurons but Hyperpolarized Pyramidal Neurons in the BLA

**Figure 3:**
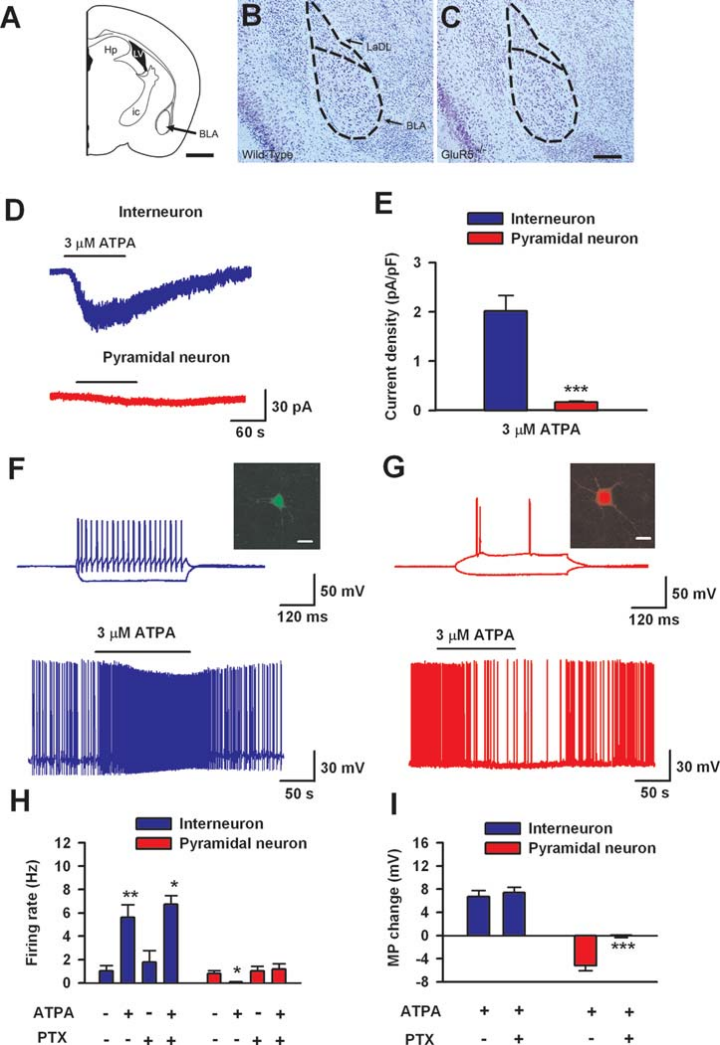
Activation of GluR5 increased neuronal excitability in interneruons but decreased excitability in pyramidal neurons. (A) Schematic organization of a parasagittal hemisection through a mouse brain depicting the approximate anatomical location where immunocytochemistry was conducted. (B and C) Nissl-stained coronal section through the BLA of representative wild-type (B) and GluR5^−/−^ (C) mice. No obvious anatomical differences between these two strains were detected. (D and E) ATPA (3 µM)-induced current in interneurons (n = 5) is significantly larger than that observed in pyramidal neurons (n = 6). (F) Interneurons were identified by their morphology and firing properties. Lucifer yellow (0.1%) was loaded through the patch pipette and confocal images were obtained after recording. A representative interneuron is shown in the inset (scale bar, 20 µM). When injected with current steps that ranged from −100 pA to 100 pA within 400 ms, interneurons showed fast spiking properties (top trace). In the same neuron, bath application of ATPA (3 µM) induced neuronal depolarization and firing of the interneuron (lower trace). Resting membrane potential for this neuron is −52.3 mV and holding current is 18.5 pA. (G) Pyramidal neurons showed different firing properties from those observed in interneurons after current injection (top trace; representative shown in the inset, scale bar, 20 µM). In the same neuron, bath-application of ATPA (3 µM) induced hyperpolarization and reduced the neuronal firing (lower trace). Resting membrane potential for this neuron was −66.6 mV and the holding current was 66.9 pA. (H) Pooled data showed that ATPA (3 µM) increased firing rate of interneurons (n = 6) while decreased that of pyramidal neurons (n = 5). Bath application of picrotoxin (100 µM) completely blocked the effect of ATPA in pyramidal neurons but not in interneurons. (I) Bath application of picrotoxin (100 µM) completely abolished the hyperpolarization in pyramidal neurons (n = 5) but not depolarization in interneurons (n = 5) induced by ATPA (3 µM). * P<0.05, **P<0.01.

What is the possible mechanism for the contribution of KA GluR5 receptors to behavioral anxiety? Electrophysiological recordings in a brain slice preparation were performed to answer this question. Previous studies of KA receptors in the amygdala were mostly performed in rats [22], [27] only few studies were performed in adult mice. We first determined that there were no obvious differences in the anatomical features and neuronal density of the amygdala between wild-type and GluR5^−/−^ mice (wild-type, 1064 ± 49 cells/mm^2^; GluR5^−/−^, 1041±50 cells/mm^2^; P = 0.28) (Figure 3 A–C). Next, we used the whole-cell patch clamp recording technique to measure GluR5 KA receptor-mediated currents in pyramidal and interneurons in the BLA. Interneurons and pyramidal neurons were identified by their different firing patterns and morphology [28] (Figure 3F and G; Table 1). Puff-applied ATPA (3 µM), the selective GluR5 agonist, induced inward currents in interneurons but small or undetectable currents in pyramidal neurons (Figure 3D and E). Significant differences were found in the ATPA-induced current densities when comparing interneurons and pyramidal neurons (interneuron: 2.0±0.3 pA/pF, n = 5; pyramidal neurons: 0.17±0.03 pA/pF, n = 6; P<0.01) (Figure 3E). In the presence of GluR5 antagonist, LY293558 (30 µM), ATPA did not induce inward current in either interneurons (n = 7) or pyramidal neurons (n = 7). Consistently, no ATPA-induced current was observed in both types of neurons in GluR5^−/−^ mice (interneurons, n = 8; pyramidal neurons, n = 6). These results indicate that ATPA selectively activated interneuronal GluR5-containing KA receptors in the BLA.

**Table 1:**
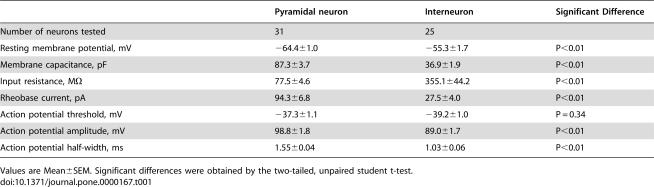
Basic electrophysiological properties of BLA neurons.

	Pyramidal neuron	Interneuron	Significant Difference
Number of neurons tested	31	25	
Resting membrane potential, mV	−64.4±1.0	−55.3±1.7	P<0.01
Membrane capacitance, pF	87.3±3.7	36.9±1.9	P<0.01
Input resistance, M*Ω*	77.5±4.6	355.1±44.2	P<0.01
Rheobase current, pA	94.3±6.8	27.5±4.0	P<0.01
Action potential threshold, mV	−37.3±1.1	−39.2±1.0	P = 0.34
Action potential amplitude, mV	98.8±1.8	89.0±1.7	P<0.01
Action potential half-width, ms	1.55±0.04	1.03±0.06	P<0.01

Values are Mean±SEM. Significant differences were obtained by the two-tailed, unpaired student t-test.

To examine the effects of ATPA on neuronal excitability and spike responses, current clamp recordings were performed. We found that ATPA produced opposite effects on interneurons and pyramidal cells. For interneurons, ATPA (3 µM) produced profound depolarization (6.7±1.1 mV) and increased firing rate (from 1.0±0.5 to 5.6±1.1 Hz; n = 6, P<0.01); while ATPA hyperpolarized the membrane (−5.2±0.9 mV, n = 5) and reduced firing rate of pyramidal cells (from 0.79±0.24 to 0.06±0.04 Hz; n = 5, P<0.05) (Figure 3F–I). Pharmacological antagonism or genetic deletion of GluR5 blocked ATPA effect in either interneurons (LY293558, from 0.95±0.49 to 0.94±0.53 Hz, n = 5, P = 0.90; GluR5^−/−^, from 1.1±0.3 to 1.2±0.4 Hz, n = 6, P = 0.86) or pyramidal neurons (LY293558, from 0.88±0.16 to 0.90±0.16 Hz, n = 7, P = 0.77; GluR5^−/−^, from 1.0±0.2 to 1.0±0.3 Hz, n = 5, P = 0.99). Considering less GluR5 currents were found in pyramidal cells, one explanation of the effects of ATPA on pyramidal cells is through GABA released from interneurons. We thus repeated these experiments in the presence of the GABA_A_ receptor antagonist, picrotoxin (100 µM). Indeed, the effect of ATPA on pyramidal neurons was completely blocked (Figure 3 H and I).

### Activation of GluR5 Enhances Spontaneous GABAergic, but not Glutamatergic Neurotransmission, in the Basolateral Amygdala

**Figure 4:**
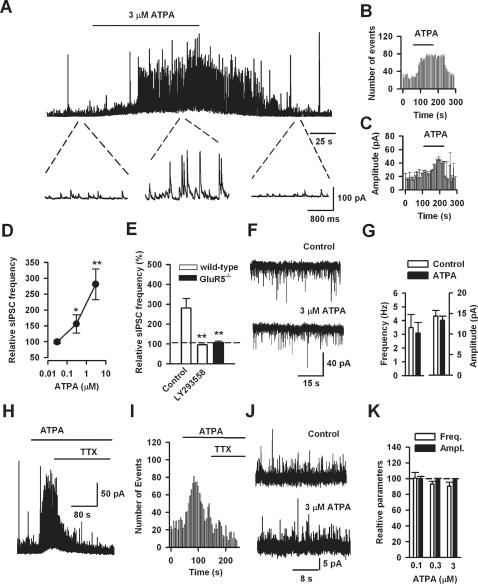
Activation of GluR5 by ATPA reversibly increased sIPSCs but not sEPScs or mIPSCs. (A) A representative example of ATPA (3 µM) modulation of sIPSCs in a BLA pyramidal neuron. The top trace represents sIPSCs recorded before, during and after ATPA application. The bottom 3 traces are presented at an expanded scale. (B and C) Time course for the ATPA-induced enhancement of sIPSC frequency and amplitude in the neuron shown in (A). Note the effect of ATPA is reversible. (D) The facilitatory effect of ATPA on sIPSC frequency is concentration dependent (0.03 µM, n = 6; 0.3 µM, n = 5; 3 µM, n = 7). (E) The effect of ATPA (3 µM) could be blocked by LY293558 (30 µM, n = 4) or in GluR5^−/−^ mice (n = 4), suggesting that the ATPA's action is mediated by GluR5. *Indicates a significant difference from control without treatment of ATPA. (F and G) ATPA (3 µM) had little effect on either frequency or amplitude of sEPSCs (n = 5). (H and I) A representative trace (F) and time course of drug effect (G) showing that TTX (1 µM) fully reversed the enhancement of sIPSC induced by ATPA treatment (3 µM). Similar results were obtained from an additional 4 neurons. (J and K) In the presence of TTX (1 µM), ATPA did not affect either amplitude or frequency of mIPSCs. Three concentrations of ATPA were used and none had significant effects on sIPSC frequency or amplitude (0.1 µM, n = 7; 0.3 µM, n = 6; and 3 µM, n = 7).

As ATPA induced firing of interneurons, we speculated that activation of GluR5 would affect GABA release in the BLA. To test this idea, we studied the effect of ATPA on GABAergic transmission. At a holding potential of 10 mV and in the presence of AP-5 (50 µM) and GYKI53655 (100 µM), a selective AMPA antagonist, spontaneous inhibitory postsynaptic currents (sIPSCs) were pharmacologically isolated in putative pyramidal neurons in BLA slices from adult mice. Upon bath application of ATPA (3 µM), the frequency of sIPSCs was significantly increased to 281.3±48.4% of control (from 6.1±0.7 Hz to 17.1±2.0 Hz, n = 7, P<0.01) (Figure 4A and B). The mean amplitude of sIPSCs also significantly increased (325.4±85.1%, n = 7, P<0.01) (Figure 4A and C). This facilitation was reversible after the washout of ATPA. Moreover, the effect of ATPA was concentration-dependent, with no significant effect for 0.03 µM, a moderate but significant effect for 0.3 µM and a robust increase by 3 µM ATPA applied to the recording bath (Figure 4D). The facilitatory effect of ATPA (3 µM) was completely abolished in the presence of selective GluR5 antagonist, LY293558 (30 µM, n = 4), or in the GluR5^−/−^ mice (n = 4) (Figure 4E).

We next tested if glutamatergic neurotransmission in the BLA was affected by ATPA application. At a holding potential of −70 mV and in the presence of PTX (100 µM), spontaneous excitatory postsynaptic currents (sEPSCs) were pharmacologically isolated. We found that bath application of ATPA (3 µM) had no significant effect on either the frequency or amplitude of sEPSCs in BLA pyramidal neurons (n = 5, Figure 4F and G).

### Increase of GABA Release by ATPA is Action Potential Dependent

Previous studies conducted in the hippocampus and cortex have demonstrated that KA depolarizes GABAergic interneurons via activation of somatodendritic KA receptors [29]–[32] while work in the rat spinal dorsal horn [33]–[35] and BLA [25] suggests that the effect of KA and ATPA are mediated by the presynaptic location of KA receptors. To investigate whether somatodendritic or presynaptic KA receptors are involved in regulating GABA release in the BLA of adult mice, we conducted experiments where tetrodotoxin (TTX, 1 µM) was applied to the recording bath. We found that the facilitatory effect of ATPA on sIPSCs was completely reversed by blockade of sodium channels with TTX (Figure 4H and I, n = 5). This finding suggests that the effect of ATPA is dependent on action potential discharge. To further confirm these results, we studied the effects of ATPA on the frequency and amplitude of miniature inhibitory postsynaptic currents (mIPSCs) in the presence of TTX. Three concentrations of ATPA (0.03, 0.3 and 3 µM) were tested and none of them had a significant effect on either the frequency or amplitude of mIPSCs (Figure 4J and K). Taken together, these results demonstrate that effects of ATPA were not due to an enhancement of presynaptic GABA release or postsynaptic GABA_A_ receptor function, but rather associated with triggering action-potentials in interneurons.

### Endogenous Activation of GluR5 Enhanced GABAergic Transmission and Tonic GABA Current in the BLA

**Figure 5:**
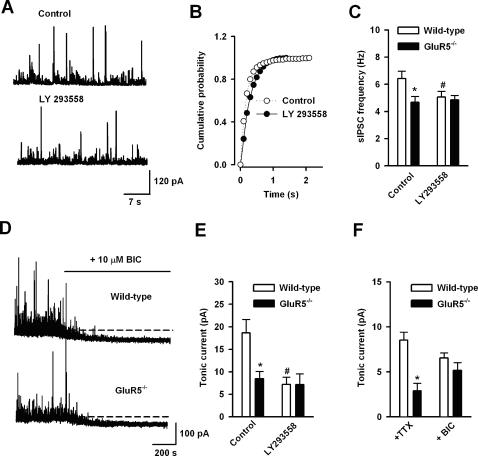
Endogenous activation of GluR5 increases GABA release and tonic GABA current in the BLA. (A) Representative traces of LY293558 (30 µM) modulation of sIPSCs in a BLA pyramidal neuron. (B) Cumulative probability plot showing that LY293558 application decreased sIPSC frequency in the neuron showed in (A). (C) Pooled data showing that LY293558 reduced the frequency of sIPSCs in wild-type mice. GluR5^−/−^ showed the decreased sIPSC frequency compared that in wild-type mice. * P<0.05 compare with GluR5^−/−^ mice, # P<0.05 compared with control without LY293558 application in wild-type mice. (D) Perfusion of bicuculline (10 µM) induced a baseline shift (the tonic GABA current) in BLA neurons from both wild-type and GluR5^−/−^ mice. (E) Tonic GABAergic current was reduced in BLA pyramidal neurons of GluR5^−/−^ mice (wild-type, n = 12; GluR5^−/−^, n = 11). In the presence of LY293558 (30 µM), tonic GABA current was also significantly reduced (n = 9). * P<0.05 compare with GluR5^−/−^ mice, # P<0.01 compared with control without LY293558 application in wild-type mice. (F) Bath application of TTX (1 µM) induced tonic GABAergic current in wild-type mice (n = 8). In GluR5^−/−^ mice (n = 8), the TTX-induced tonic current is smaller than that in wild-type mice. Further application of bicuculline caused the similar tonic current in both mice. * P<0.05.

To further explore whether there is endogenous activation of GluR5 in the BLA, we studied sIPSCs and mIPSCs after pharmacological blockade or genetic deletion of GluR5. We found that bath-applied LY293558 (30 µM) significantly reduced the sIPSC frequency in BLA pyramidal neurons of wild-type mice (control, 6.3±0.6 Hz; in LY293558, 5.0±0.4 Hz n = 6, P<0.05, Figure 5A–C). However, we found no effect of LY293558 on mIPSC frequency (control, 2.5±0.5 Hz; in LY293558, 2.2±0.4 Hz, n = 6; P>0.05). Next, we compared sIPSCs in wild-type and GluR5^−/− ^mice. A significant difference in sIPSCs frequency was detected between wild-type and GluR5^−/− ^animals (wild-type, 6.4±0.5 Hz, n = 12; GluR5^−/−^, 4.7±0.4 Hz, n = 11; P<0.05) (Figure 5C). In GluR5^−/−^ mice, LY293558 has no more effect on sIPSCs (Figure 5C). Surprisingly, we found that the frequency of mIPSCs in the BLA of GluR5^−/−^ mice was significantly decreased when compared to that of wild-type mice (wild-type, 1.9±0.3 Hz, n = 15; GluR5^−/−^, 1.1±0.3 Hz, n = 13; P<0.05). These results suggest that endogenous activation of GluR5 would facilitate GABAergic transmission.

Presynaptic GABA release participates in the generation of tonic GABA current, which is important for the neuronal excitability [36], [37]. Tonic GABA currents in the BLA neurons were revealed after the application of a selective competitive GABA_A_ receptor antagonist, bicuculline (10 µM) in wild-type mice (Figure 5D and E, −18.6±2.9 pA, n = 12). In GluR5^−/−^ mice, tonic GABA currents were significantly decreased (Figure 5D and E, −8.5±1.6 pA, n = 11, P<0.05). Tonic GABA currents were also significantly reduced in the presence of LY293558 in wild-type and GluR5^−/−^ mice (wild-type, 7.2±1.6 pA, n = 9, P<0.01; GluR5^−/−^, 7.1±2.4 pA, n = 8, P<0.05, Figure 5E). Both vesicular and non-vesicular releases of GABA contribute to tonic receptor activation [38], we want to know whether GluR5 modulates different sources of GABA for tonic GABA current. Bath application of TTX (1 µM) revealed the tonic inward current in the wild-type mice (8.5±0.9 pA, n = 8) and subsequent application of bicuculline (10 µM) caused increase of tonic GABA current (6.5±0.9 pA, n = 8, Figure 5F). In GluR5^−/−^ mice, we found that TTX-sensitive tonic current is significantly decreased (2.9±0.8 pA, n = 8, P<0.05, Figure 5F). The further application of bicuculline induced the similar inward current in wild-type and GluR5^−/−^ mice (5.2±0.9 pA, n = 8, P = 0.43, Figure 5F). These results suggest that endogenous interneuronal GluR5 activation modulates action potential-dependent tonic GABA current.

### Activation of GluR5 in the Basolateral Amygdala Inhibits Excitatory Projections to the Central Amygdala

**Figure 6:**
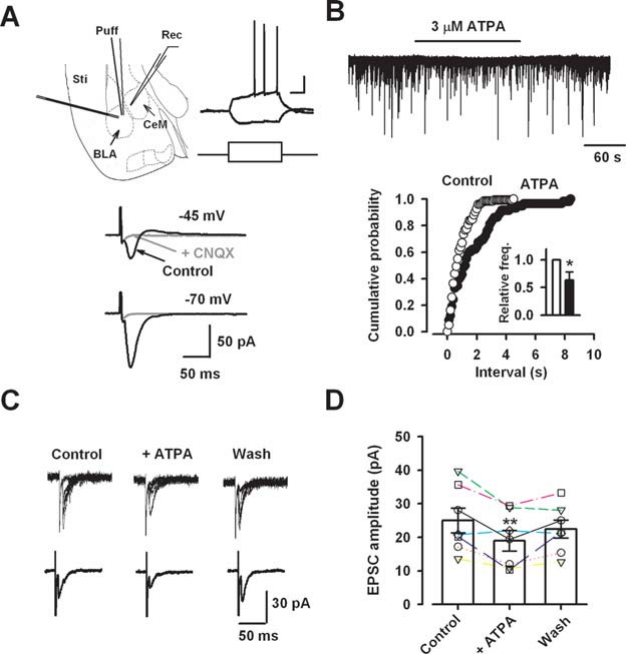
Activation of GluR5 in the BLA affected the output to the CeM in the amygdala. (A) Diagram showing the placement of the stimulating electrode (Sti) and drug puff-application pipette (Puff) in the BLA as well as recording electrode (Rec) in the CeM (left). A typical late-firing neuron showed the delayed firing properties in respond to current steps that from −50 pA to 75 pA within 400 ms (right). Resting membrane potential for this neuron is −63.3 mV. Scale bar, 15 mV and 80 ms. Lower traces showing evoked responses in a CeM neuron by electric stimulation in the BLA. At holding potential of −45 mV, biphasic responses were observed and CNQX application could block both of them. At holding potential of −70 mV, only inward current was observed. (B) Typical recording and cumulative probability plot showing that ATPA (3 µM) application in the BLA decreased sEPSCs in a CeM late-firing neuron. Inset: Relative frequency (freq.) during ATPA application was significantly decreased (n = 6). (C) Typical traces showing that after puff application of ATPA (3 µM) in the BLA, eEPSCs recorded in a CeM neuron were decreased. (D) Individual experiments and statistical results showing that ATPA reduced eEPSC amplitude (n = 7, each neuron represented by a separate symbol). * P<0.05, **P<0.01.

The inhibitory drive from local inhibitory neurons targeting to pyramidal neurons is critical for regulating neuronal excitability and subsequent information processing in the brain [39], [40]. Since there are synaptic connections from the BLA to the CeM [41], [42], we wanted to determine if GluR5 modulation on GABAergic transmission affects synaptic projection within this pathway. To delineate the role of GluR5 in the transmission of information from the BLA to the CeM, we locally puff-applied ATPA to the BLA and measured spontaneous excitatory post-synaptic currents (sEPSCs) in CeM neurons (Figure 6A). Most CeM neurons showed delayed firing properties (Figure 6A). Application of ATPA in the BLA significantly reduced the frequency of sEPSCs in CeM neurons (63.3±15.2%, n = 11, P<0.05, Figure 6B).

Next, we measured the effect of GluR5 activation on evoked EPSCs (eEPSCs) in CeM neurons obtained by stimulating the BLA. These responses were polysynaptic, showing both inward glutamatergic and outward GABAergic currents at a holding potential of −45 mV (Figure 5A). Application of CNQX (20 µM) blocked both inward and outward responses, confirming that the outward GABAergic current was due to feed forward inhibition from the intercalated cell masses, an inhibitory interface between BLA and CeM [42], [43]. At holding potential of −70 mV, which is near the reversal potential for GABAergic currents, only inward currents were observed, which could be completely abolished by CNQX (Figure 6A). After obtaining a stable eEPSC, ATPA (3 µM) was locally applied to the BLA. Puff-applied ATPA into the BLA significantly and reversibly decreased eEPSCs in the CeM (75.7±6.3%, n = 7, P<0.01, Figure 6C and D). These findings indicate that the activation of GluR5 in the BLA exerts an inhibitory effect on excitatory output to the CeM in the amygdala.

### Role of GluR5 in Excitatory Synaptic Transmission and Plasticity from the Basolateral Amygdala to Central Amygdala

**Figure 7:**
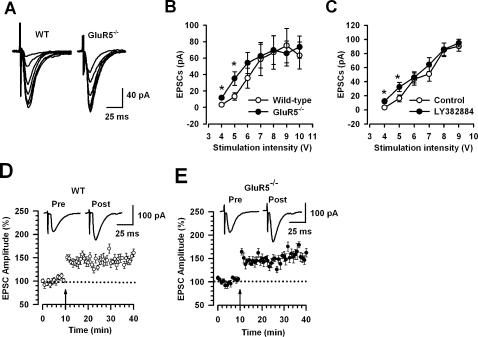
GluR5^−/−^ mice exhibit less synaptic efficacy from the BLA to CeM, but no change long-term potentiation. (A) Typical samples showing eEPSCs recorded in CeM neurons by stimulation in the BLA in wild-type and GluR5^−/−^ mice. The stimulation intensities range from 4 V to 10 V. (B) Input-output curve of eEPSCs indicated that at lower stimulation intensity, eEPSCs in wild-type mice were significantly smaller than those in GluR5^−/−^ mice. No difference was found at a higher stimulation intensity (wild-type, n = 8; GluR5^−/−^, n = 7). (C) Local application of LY382884 (10 µM) has similar effect on input-output curve of eEPSC to that in GluR5^−/−^ mice, increasing weak stimulation-induced eEPSCs (n = 8). (D and E) LTP could be induced in CeM neurons by theta burst stimulation in the BLA from wild-type mice (n = 8) or GluR5^−/−^ mice (n = 8). There was no significant difference in potentiation between groups. The dashed line indicates the mean basal synaptic responses and the arrow shows the time point of LTP induction. *P<0.05.

To further address the role of GluR5 in the regulation of intra-amygdaloid connections, we compared baseline synaptic transmission in BLA to CeM synapses between wild-type and GluR5^−/−^ mice. At a holding potential of −70 mV, evoked EPSCs in CeM neurons were obtained by stimulating the BLA. First, we tested the input-output relationship of AMPA receptor-mediated eEPSCs in CeM neurons. In the presence of AP5 (50 µM), we found a significant difference in the amplitude of eEPSCs between wild-type and GluR5^−/−^ mice at lower intensities of stimulation (4V, 3.1±1.3 pA, n = 8 vs 11.8±2.2 pA, n = 7, P<0.05; 5V, 13.4±4.6 pA, n = 8 vs 35.2±8.4 pA n = 7, P<0.05, Figure 7A and B). However, when the stimulation intensity was increased, no difference was found in the eEPSCs between control and knockout mice (Figure 7A and B). To further study the selective role of GluR5 in the BLA, we locally puffed selective GluR5 antagonist, LY382884 (10 µM) into the BLA and then studied the input-output curve in wild-type mice. Consistently, we found that application of LY382884 in the BLA increased eEPSCs in CeM at lower stimulation intensity (Figure 7C, n = 8).

In addition to basal synaptic transmission, we also examined whether the GluR5 deletion affected long-term synaptic potentiation in the CeM. It has been reported that LTP in BLA to CeM synapses could be induced by high frequency stimulation [44]. We used theta burst stimulation, a strong LTP induction protocol previously used in the anterior cingulate cortex and lateral amygdala, to induce LTP in CeM neurons [45], [46].?A significant, long-lasting synaptic potentiation was observed in slices of wild-type mice after induction (150.2±5.9% of baseline, n = 8 slices/6 mice, P<0.05 compared with baseline responses before induction, Figure 7D). This protocol also induced LTP in GluR5^−/−^ mice (157.4±8.0%, n = 8 slices/5 mice; P<0.05 compared with baseline responses, Figure 7E) and the magnitude of synaptic potentiation in GluR5^−/−^ mice was similar to that of wild-type mice (P<0.05). These results indicate that long-term plasticity was normal in BLA to CeM synapses in GluR5^−/−^ mice.

## Discussion

The present work provides strong evidence for a role of GluR5-containing KA receptors in anxiety. Our results show that activation of GluR5 potently regulates GABAergic transmission and network excitability in the amygdala. Based on these findings we propose that impaired GABAergic transmission, due to the lack of GluR5, may underlie the development of anxiety-like behavior in GluR5^−/−^ mice. Manipulations directed at GluR5-containing receptors may aid in the development of selective and effective anxiolytic drugs in the future.

Despite an increased understanding of the physiological actions of GluR5 at the cellular level, less is known about its role in behavioral functions [19]. Recent studies showed that GluR5 is involved in chronic pain [47], [48] and epileptogenesis [27], [49], [50]. However, a role for GluR5 in anxiety, an emotional behavior related to amygdala function, has not been reported. In the present study, our results clearly demonstrate a role for GluR5 in anxiety. GluR5^−/−^ mice showed an increase in anxiety-like behavior in both the elevated plus maze and light/dark test. These changes are unlikely due to a reduction in general locomotion since performances in the open field and on the RotaRod were similar between knockout and wild-type mice. As further demonstrations of the role of GluR5 in anxiety, we found that wild-type mice displayed less anxious behavior when receiving ATPA whereas they showed more anxiety by local GluR5 antagonism in the BLA. We were not able to collect data after microinjection of ATPA into the amygdala due to the occurrence of side effects. These novel findings raise the possibility that GluR5 in the BLA may underlie the behavioral expression of anxiety. Although our microinjection results clearly showed that GluR5 in the BLA is involved in the expression of anxiety, we cannot exclude the possibility that other brain regions may also contribute to the increased anxiety phenotype in GluR5^−/−^ mice. In addition, the evidence that EPM involves the amygdaloid complex is weak, despite that recent studies do show that amydala is important for the anxious behaviors. For example, intra-amygdala injection of different metabotropic glutamate receptor agonists decreased anxious behavior in the EPM [51] and anxiogenic drugs lead to an increase in immediate early gene expression within the amygdale [52].

Previous reports show that GluR5 is expressed in the BLA where it participates in synaptic transmission, modulation and plasticity in young rats [22], [25]. The present study provides direct evidence for the important role of GluR5-containg KA receptors in inhibitory interneurons. Furthermore, the current density of GluR5 activation was larger in interneurons as compared to pyramidal neurons. In agreement with these results we found that ATPA induced interneuronal depolarization and spiking behavior, in contrast to pyramidal neurons where ATPA triggers hyperpolarization. We also showed that GABA_A_ receptor antagonist picrotoxin blocked hyperpolarization in pyramidal neurons, but not the depolarization in interneurons. Taken together, these results lead us to conclude that: (1) GluR5 is predominant and highly functional in interneurons; activation of GluR5 in this cell type triggers neuronal firing and subsequent release of GABA; (2) Significantly less GluR5 activity (or expression) is found in pyramidal neurons as compared to levels in interneurons; (3) GluR5 activation in pyramidal neurons may be counteracted by the activation of GABA_A_ receptors, resulting from the bulk release of GABA from interneurons which were fired by GluR5 activation. Therefore, activation of GluR5 reduces the excitability of pyramidal cells in the BLA.

Our result showed that GluR5 is selectively acting on inhibitory but not excitatory synaptic transmission in the BLA. Bidirectional modulation of GABA release by presynaptic GluR5 (measured by both mIPSCs and eIPSCs) was reported in the young rat BLA, in which different types of GluR5-containing KA receptors, with different agonist affinities, were proposed to mediate the biphasic effect [25]. Our results, using adult mice, suggest a different mechanism. We first showed that, ATPA did not affect the frequency or amplitude of mIPSCs. Three different concentrations of ATPA were tested and none of them had modulatory effects on mIPSCs, although highly visible impacts on sIPSCs were observed. Our results suggest that GluR5 may be located on somatodendrites rather than on presynaptic terminals. At present, no clear explanations exist for these differences. However, it may be possible that the age (young vs. adult animals) and the species used (mouse vs. rat) may contribute to these differences.

Two lines of evidence in the present study directly showed that GluR5 is endogenously activated. First, the frequency of sIPSCs and mIPSCs was significantly lower in GluR5^−/−^ mice compared to wild-type mice. Second, the selective GluR5 antagonist, LY293558 reduced sIPSC frequency. Therefore, we believe that GluR5 is tonically activated by endogenous glutamate in the extracellular fluid. In addition to synaptic currents (phase current), GABA_A_ receptors also mediate a persistent tonic current, which reflects the activation of high-affinity GABA_A_ receptors by low ambient GABA concentration [38]. It has been reported that tonic GABA current is mainly mediated by α5-containing GABA_A_ receptors in hippocampal CA1 neurons [53] and by δ-containing GABA_A_ receptors in granule cells of hippocampal dentate gyrus or cerebellum [15]. However, no report is available concerning the tonic inhibitory currents in amygdala neurons. This is the first study to report that GABA_A_ receptor mediated tonic inhibition exists in pyramidal neurons in the amygdala of adult mice. Furthermore, we showed that this inhibition is under the tonic regulation of GluR5-containing KA receptors on BLA interneurons. Genetic deletion of GluR5 or pharmacological blockade of GluR5 could significantly reduce the tonic GABA current. Our results showed that TTX itself induced tonic GABA current in WT mice but significant smaller tonic current in GluR5^−/−^ mice, while subsequent application of bicuculline caused similar tonic current in both. These results suggest that the source of GABA for tonic GABA current partially involves the action potential-dependent GABA release, which may spill over to activate the extrasyanptic GABA_A_ receptors.

Tonic inhibition is thought to contribute to the control of neuronal excitability by reducing neuronal input-output functions [38]. Consistently, we found a reduced input-output relationship of synaptic transmission from the BLA to the CeM in GluR5^−/−^ mice. The increased synaptic efficacy observed at low, but not high, intensities of stimulation suggests that GluR5 finely modulates synaptic transmission by tonic inhibition. Local application of selective GluR5 antagonist, LY382884 in the BLA mimicked the phenoma found in GluR5^−/−^ mice, suggesting that endogenous activation of GluR5 in the BLA may modulate the functional output to the CeM. Local application of ATPA (3 µM) in the BLA still decreased strong stimulation-induced eEPSCs recorded from the CeM (control, 81.2±12.1 pA; ATPA, 44.0±6.6 pA, n = 6, P<0.05). These results suggest that exogenous activation of GluR5 in the BLA has robust inhibitory effect while endogenous activation of GluR5 exert defined modulatory effect on projection from BLA to CeM. However, we cannot exclude the possibility that the evoked responses may partially contain that from the passing fibers.

In summary, we report here that GluR5^−/−^ mice showed increased anxiety-like behavior and the mechanisms for GluR5's role in anxiety may involve impaired GABA release, tonic inhibition, network excitability and the excitatory output from BLA to CeM. However, it should be noted that the anxiety phenotype detailed here was obtained in a global knockout for GluR5, which lacks regional specificity. It is therefore not possible to discount potential roles for GluR5 in other brain regions related to emotional behavior. Our results using local administration of a selective GluR5 antagonist to show the same increase in anxious behavior partially addresses this concern. However, further studies, using temporally and spatially selective gene targeting techniques, are needed to demonstrate the role of amygdalar GluR5 in anxiety in a region specific manner void of possible developmental compensations. Despite this limitation, our results clearly show that GluR5-containing KA receptors are involved in regulating anxious behavior and may serve as a target for novel anxiolytics in the future.

## Materials and Methods

### Animals

Adult C57BL/6 mice were purchased from Charles River (6–10 weeks old). GluR5^−/−^ mice were obtained as gifts from Dr. Stephen Heinemann (Salk Institute, San Diego, CA) [54], [55]. GluR5^−/−^ mice were maintained on a mixed 129Sv x C57BL/6 background and wild-type littermates were used as controls. As it was impossible visually to distinguish mutant mice from wild-type mice, all experiments were performed in a blind fashion. All mice were maintained on a 12 h light/dark cycle with food and water provided *ad libitum*. All protocols used were approved by The Animal Care and use Committee at the University of Toronto and conform to NIH guidelines.

### Whole-cell Patch Clamp Recordings

Adult male mice (6–10 weeks old) were anesthetized with 1–2% halothane and decapitated. Coronal slices of the amygdala (300 µm) were prepared using routine methods used in our lab [48]. Slices were then transferred to a room temperature submerged recovery chamber with oxygenated (95% O_2_ and 5% CO_2_) solution containing (in mM): NaCl, 124; NaHCO_3_, 25; KCl, 2.5; KH_2_PO_4_, 1; CaCl_2_, 2; MgSO_4_, 2; glucose, 10. After a one-hour recovery period, slices were placed in a recording chamber on the stage of an Axioskop 2FS microscope (Zeiss, Thornwood, NY) equipped with infrared DIC optics for patch clamp recordings. Postsynaptic currents were recorded with an Axon 200B amplifier (Molecular Devices, Union City, CA). Stimulations were delivered using a bipolar tungsten stimulating electrode locally placed in the BLA. Electric square-wave voltage pulse (200 µs, 4–14 V) was generated using a Grass S88 stimulator (Grass instrument Co., Quincy, MA) attached to a Grass SIU5D isolator unit. Recording electrodes (2–5 M*Ω*) contained an internal solution composed of (in mM): Cs-gluconate, 120; NaCl, 5; MgCl_2_ 1; EGTA, 0.5; Mg-ATP, 2; Na_3_GTP, 0.1; HEPES, 10; pH 7.2; 280–300 mOsmol. Cs-gluconate was replaced by equimolar K-gluconate when we performed current clamp recordings. Unless stated otherwise, the membrane potential was held at −70 mV throughout all experiments. Theta burst stimulation was used as LTP induction paradigm. This protocol involved five trains of burst with four pulses at 100 Hz, at 200 ms interval; repeated four times at intervals of 10 s. When recording GABA_A_ receptor-mediated currents, a holding potential of 10 mV was used, as indicated in the body of the text.

### Immunohistochemistry

Animals (n = 6) were overdosed with sodium pentobarbital and perfused transcardially with 20 ml of 0.1M phosphate buffered saline (PBS; pH = 7.4) and 4% paraformaldehyde in PBS. Brains were then dissected out, cryoprotected in 30% sucrose, included in embedding medium (Tissue-Tek; Sakura Finetek, Torrance, CA), fast-frozen in dry-ice, cut coronally on a cryostat (20 µm), thaw-mounted on glass slides and allowed to dry overnight. Sections were re-hydrated by incubation in alcohol solutions of decreasing concentrations (100, 95, 70, 50%; 2 mins each) and placed in distilled water for 5 mins. Next, sections were placed in a 0.5% Cresyl violet solution for 5 mins, dehydrated in a series of alcohols, defatted in xylenes and coverslipped. The neuronal density was estimated using the Nissl-stained sections. Three areas around 0.01 mm^2^ in the BLA were randomly picked and number of neurons was counted in each area. The neuronal density in the slice was obtained by averaging the density in three areas.

### Pharmacological Drugs

AP-5 and GYKI53655 were used to selectively block NMDA receptors and AMPA receptors, respectively. ATPA was used to selectively activate GluR5-containing KA receptors. All chemicals and drugs were obtained from Sigma (St. Louis, MO), except for ATPA, which was purchased from Tocris (Ellisville, MO). GYKI53655 was a kind gift from Drs. John F. MacDonald (University of Toronto) and Geoffrey T. Swanson (University of Texas). Most drugs were applied to the perfusion solution using methodologies previously described by our group. Local puff application of drug was described previously [56].

### Elevated Plus Maze

The elevated plus maze (Med Associates, St. Albans, Vermont) consisted of two open arms and two closed arms situated opposite to each other. For each test, individual animals were placed in the center square and allowed to move freely for five minutes. The number of entries and time spent in each arm was recorded. ATPA (1.0, 5.0 or 10.0 mg/kg) (i.p., 0.9% saline, Sigma Aldrich, St Louis, MO) was injected 30 min before testing. Saline injections were used are controls. A video camera tracking system (Ethovision, Noldus VA) was used to generate the traces shown in Figure 1D.

### Open Field Activity Monitor

To record horizontal locomotor activity we used the Activity Monitor system from Med Associates (43.2×43.2×30.5 cm; MED-associates, St. Albans, VT). Briefly, this system uses paired sets of photo beams to detect movement in the open field and movement is recorded as beam breaks. The open field is placed inside an isolation chamber with dim illumination and a fan. Each subject was placed in the center of the open field and activity was measured for 30 minutes.

### Light/Dark Test

The testing apparatus (Med Associates, St Albans, VT) consisted of a rectangular Plexiglas box (44×8.5×25 cm) equally divided into a light compartment connected by a door (17 cm in height) to a dark compartment. Each mouse was placed in the light box and was allowed 10 sec to explore before the door to the dark box was opened. The time spent in both compartments was recorded for 10 min.

### RotaRod

To test for motor coordination we used the RotaRod from Med-Associates (St, Albans, Vermont). One hour before testing, animals were trained on the RotaRod set at 16 rpm until they could stay on for 30 s. For testing, the RotaRod was set to accelerate from 4 rpm to 40 rpm over the course of 5 min, the latency to complete two consecutive rolls or to drop off the rod (performance) was recorded.

### Amygdala Cannulation and Microinjection

Under ketamine and xylazine anesthesia, 24-gauge guide cannulas were implanted bilaterally into the BLA (−1.4 mm anterior to Bregma, ±3.3 mm lateral from the midline, 5.0 mm beneath the surface of the skull). Mice were given at least 2 weeks to recover after cannula implantation. All procedures were performed in accordance with the requirements of the Animal Studies Committee at the University of Toronto. The 30-gauge injection cannula was 0.1 mm lower than the guide. For intra-amygdala infusion, 0.5 µl LY382884 (4 µg/µl, 5% DMSO) or vehicle was delivered bilaterally. Twenty minutes later, mice were tested in the EPM.

### Data Analysis

Results were expressed as mean ± standard error of the mean (S. E. M.). Statistical comparisons were performed with the Student T-test or one-way analysis of variance (ANOVA). Analysis of miniature or spontaneous postsynaptic currents was performed with cumulative probability plots and was compared using the Kolmogorov-Smirnov (K-S) test for significant differences. In all cases, P<0.05 was considered statistically significant.
